# Case Report: Endoscopic ultrasound-guided fine-needle biopsy for the diagnosis of esophageal tuberculosis

**DOI:** 10.3389/fmed.2025.1575045

**Published:** 2025-10-30

**Authors:** Hailin Yang, Huajing Ke, Yupeng Lei, Yong Zhu

**Affiliations:** ^1^Queen Mary School, Nanchang University, Nanchang, China; ^2^Department of Gastroenterology, First Affiliated Hospital of Nanchang University, Nanchang, China

**Keywords:** esophagus, tuberculosis, endoscopic ultrasonography, EUS-FNB, diagnosis

## Abstract

**Aim:**

To summarise the characteristics of esophageal tuberculosis, and to evaluate the role of endoscopic ultrasound-guided fine-needle biopsy (EUS-FNB) in the diagnosis of esophageal tuberculosis.

**Methods:**

A retrospective analysis of esophageal tuberculosis patients diagnosed by the EUS-FNB between May 2012 and August 2023 was reported. Final diagnosis was based on histopathology, clinical context in combination with response to antitubercular therapy. Tissue acquisition of both esophageal lesions and enlarged mediastinal lymph nodes was performed by EUS-FNB. The variables evaluated were clinical features, location and diameter of the esophageal lesions and enlarged lymph nodes.

**Results:**

Six esophageal tuberculosis patients without surgical management were finally identified by the performance of EUS-FNB. The most common clinical feature was progressive dysphagia. The site of lesions was most common located in the middle of esophagus (66.7%). Diameter of lesions range from 1.2 to 4.1 cm. Multiple enlarged mediastinal lymph nodes were of various sizes (range 0.6–1.6 cm in diameter). CT scan revealed focal thickened esophageal wall, esophageal stenosis, and a mass with moderate and heterogeneous enhancement in five patients (83.33%), which indicating esophageal carcinoma. Purified protein derivative (PPD) and T cell spot test (T-spot) were positive in four cases (66.7%), which were significant in the diagnosis. EUS-FNB provided the pathological diagnosis of tuberculosis in four cases.

**Conclusion:**

Esophageal tuberculosis should be distinguished from advanced esophageal carcinoma and leiomyoma. Moreover, EUS-FNB is an excellent method in both image and tissue biopsy to establish an accurate diagnosis of esophageal tuberculosis.

## Introduction

In the past few decades, there has been a concerted global effort to eradicate tuberculosis, but it still accounts for a major health problem in developing countries, and these countries experience the highest morbidity and mortality rates (1, 2). Tuberculosis infection can cause multiorgan involvement and it is most often seen in the lungs, and extrapulmonary tuberculosis accounts for approximately 20% of all cases (3, 4). Esophageal tuberculosis is a very rare clinical entity, although the digestive tract is the sixth most common affected site. In autopsy, esophageal involvement was seen in only 0.04–0.2% of all tubercular patients (5). Moreover, esophageal tuberculosis is similar to esophageal carcinoma both clinically and radiologically, so the lesions may be mistaken for malignancy (6). Therefore, diagnosing esophageal tuberculosis is challenge, and most cases are misdiagnosed before surgical exploration. Endoscopic ultrasound (EUS) guided fine-needle biopsy (FNB) is performed under realtime ultrasound guidance, and it can visualize and biopsy structures beneath esophageal mucosa and beyond the esophageal wall. The purpose of this study is to summarise the characteristics of esophageal tuberculosis, and to evaluate the role of EUS-FNB in the diagnosis of esophageal tuberculosis.

## Materials and methods

This is a retrospective study of esophageal tuberculosis from May 2012 to August 2023 at a tertiary center in Wuhan, China. This study was reviewed and approved by the Ethics Committee of the Union Hospital, Tongji Medical College, Huazhong University of Science and Technology (No. 2024-0546). Since this study is a retrospective study, the informed consent form was waived. The records of all patients with a diagnosis of esophageal tuberculosis were reviewed. Patients were excluded from the study if they met the following criterions: suspected or newly diagnosed esophageal cancer; received surgical exploration. Confirmative pathological diagnosis of tuberculosis was defined by at least one of the three following criteria: caseous necrosis, positive for acid fast bacilli (AFB) on Ziehl–Neelsen stain or epithelioid granuloma with necrosis. Final diagnosis was based on histocytopatholog, clinical context in combination with response to antitubercular therapy.

Six patients were finally identified by the performance of EUS-FNB. Clinical data were analyzed, including symptoms, laboratory examinations, radiological imaging, endoscopic and endosonographic features, and histocytopathological features.

EUS was performed using a miniature ultrasonic probe (UM-2R 12 MHz, Olympus, Tokyo, Japan) with a ultrasound endoscope (Olympus, ME2, UCT260, Tokyo, Japan), and a 22-G needle (Echo-Tip Ultra, Wilson-Cook Medical Inc., Winston-Salem, USA) was used for puncture.

## Results

### Clinical features, laboratory and radiological findings

The average age of the six patients was 65.33 years (range 59–79 years), including four females (62.00 ± 6.58 years) and two males (72.00 ± 9.90 years). Four patients (66.67%) manifested as progressive dysphagia, with or without odynophagia. Two patients (33.33%) manifested as retrosternal pain. A past history of tuberculosis was found in none of the six patients. None showed signs of weight loss or fever. Four cases were positive in the tuberculosis test by both tuberculin skin test performed with purified protein derivative (PPD) and T cell spot test (T-spot). Tuberculosis antibody was positive in three cases. The results of laboratory examinations revealed that erythrocyte sedimentation rate (ESR) ranged from 5 to 38 mm/h. Blood routine examination, C-reactive protein (CRP) and liver function were all normal in six patients.

All patients underwent chest and esophagus computed tomography (CT) scan. CT scan revealed focal thickened esophageal wall, esophageal stenosis, and a mass with moderate and heterogeneous enhancement in five patients, which indicating esophageal carcinoma. Four patients with multiple mediastinal lymph nodes enlargement, and one patient with lung fibrosis. Participants’ characteristics are shown in Table 1.

**Table 1:** Clinical features and CT imaging.

No.	Sex	Age (years)	Disease duration	Symptoms	Previous history of tuberculosis	ESR (mm/h)	PPD	T-Spot	TB-Ab	Lymph nodes (cm)	Chest CT scan
1	F	63	20 days	Dysphagia	−	5	+	+	−	+ (0.6)	Esophageal wall thickening
2	F	59	3 months	Retrosternal pain	−	8	−	−	−	+ (1.4)	Esophageal wall thickening
3	F	61	1 month	Retrosternal pain	−	10	+	+	+	+ (1.1)	Esophageal wall thickening
4	M	65	15 days	Dysphagia	−	38	+	+	−	+ (1.6)	Esophageal stenosis
5	M	79	2 months	Dysphagia with odynophagia	−	15	+	+	+	−	Lung fibrosis
6	F	65	4 months	Dysphagia with odynophagia	−	9	−	−	+	−	Esophageal wall thickening

### Endoscopic features

**Figure 1:**
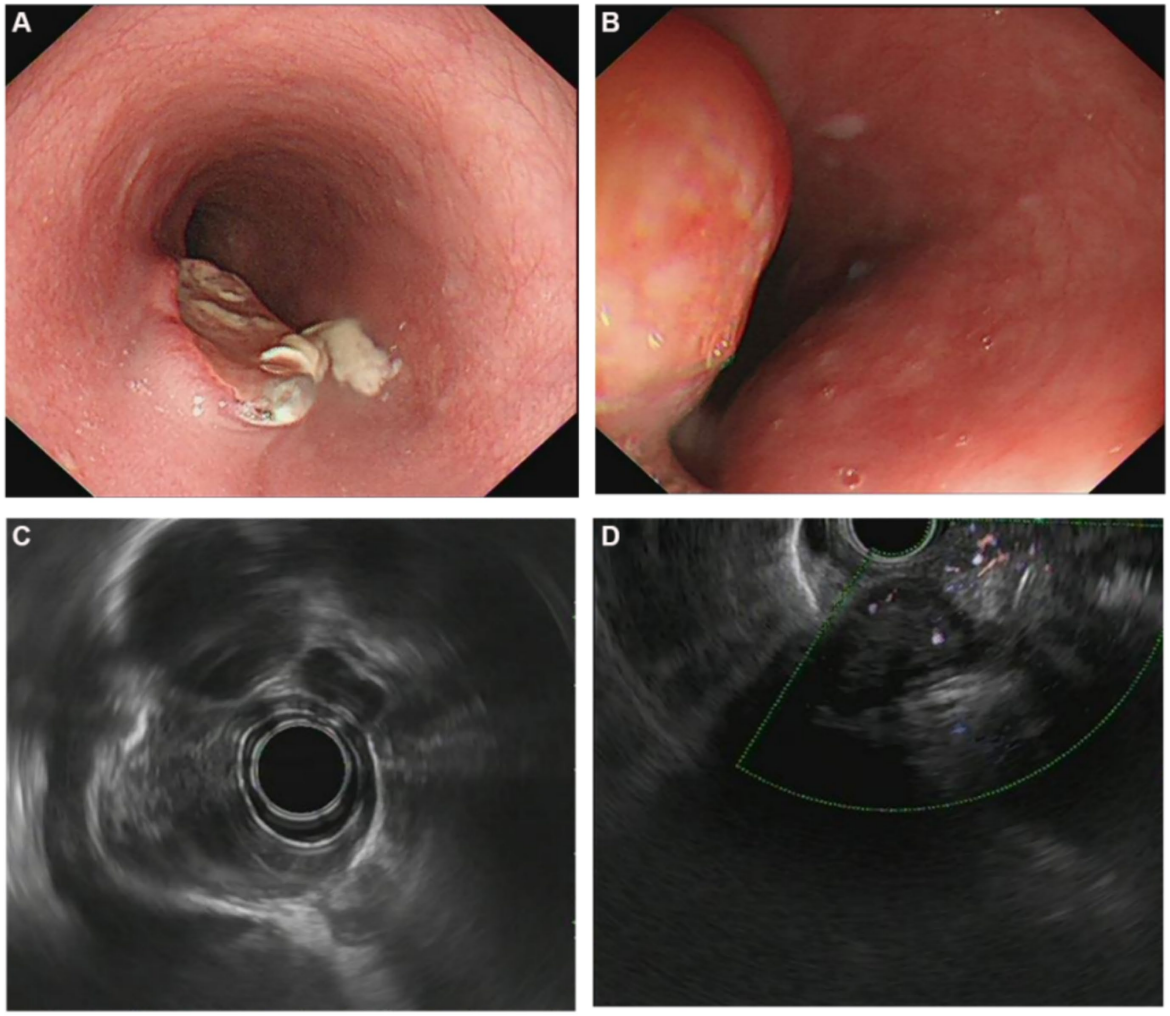
**(A)** Endoscopic and endosonographic images of patients with esophageal tuberculosis. Endoscopic image showing ovoid ulcer with yellowish-white caseous attachment at the edge; **(B)** Endoscopic image showing an esophageal protuberant focus; **(C)** Endosonographic images showing thickened esophageal wall, disappeared layer, heterogeneous and hypoechoic change; **(D)** Endosonographic images showing heterogeneous hypoechoic mass in the thickened esophageal wall, interruption and breakage of the esophageal adventitia, and fusion with mediastinal lymph nodes.

The site of lesions were located in the middle of esophagus in four patients while in another two were present in the lower esophagus. Five patients with esophageal protuberant focus. Four of them had ulcerated mucosa overlying the lesion while only one had intact mucosal surface. No extrinsic bulge but ulcers was only seen in the remaining one (Figures 1A,B). The characteristics of endoscopic features are presented in Table 2.

**Table 2:** Endosonographic features of esophageal tuberculosis.

No.	Location	Size (cm)	Lesion type	Homogeneity	Echogenicity	Layer	Adventitia	FNB
1	Mid-esophagus	1.2	Protuberant focus	Heterogeneous	Hypoechoic	Disappeared	Interrupted	Scrofula
2	Mid-esophagus	2.7	Protuberant focus	Heterogeneous	Hypoechoic	Disappeared	Interrupted	Inflammation
3	Mid-esophagus	4.1	Protuberant focus	Heterogeneous	Hypoechoic	Disappeared	Interrupted	Inflammation
4	Lower esophagus	2.0	Protuberant focus	Heterogeneous	Hypoechoic	Ambiguous	Interrupted	Tuberculosis
5	Mid-esophagus	2.4	Ulcers	Heterogeneous	Hypoechoic	Disappeared	Interrupted	Epithelioid granuloma with caseous necrosis
6	Lower esophagus	3.2	Protuberant focus	Heterogeneous	Hypoechoic	Ambiguous	Interrupted	Epithelioid granuloma with caseous necrosis

### Endosonographic features

EUS showed involvement of thickened esophageal wall by a heterogeneous and hypoechoic lesions with interrupted of the adventitia in all cases (Figures 1C,D). Diameter of lesions range from 1.2 to 4.1 cm. Disappeared layers were seen in the thickened lesions in four patients, and ambiguous layers were observed in the remaining two. Multiple enlarged mediastinal lymph nodes in close proximity to or fused with each other were seen in four patients. The lymph nodes were of various sizes which between 0.6 and 1.6 cm in diameter. Endosonographic features are summarized in Table 2.

### Histopathology/cytopathological features

EUS-FNB of both lesions and enlarged lymph nodes was performed in all cases, and it provided the pathological diagnosis of tuberculosis in four cases. Chronic granulomatous lesions with caseous necrosis was found in four, and Ziehl–Neelsen stain was positive in only one case. However, two cases showed mucosal tissues with chronic inflammatory changes, among which one was accompanied by hyperplasia and thickening of the overlying squamous epithelium and focal granuloma formation, for which the evidence was insufficient to diagnose tuberculosis. Therefore, the overall diagnostic yield of EUS-FNB is 66.67% in our study.

### Treatment and follow-up

All patients were treated with standard antitubercular therapy (isoniazid, rifampin, pyrazinamide, and ethambutol). As to the two patients whose pathological examination showed nonspecific chronic inflammation, empirical antitubercular therapy was administered based on EUS morphology. At 3 months of follow-up, the masses were significant diminution in all cases. They were sequentially followed up for 12 months, and all patients had complete resolution of dysphagia and retrosternal pain. Moreover, EUS showed resolution of esophageal lesions and lymph nodes. Thus, antitubercular treatment was successful in all patients.

## Discussion

Esophageal tuberculosis is a very rare clinical entity (7). It is usually secondary to mediastinal lymph node tuberculosis. However, primary esophageal tuberculosis is extremely uncommon due to the self-defense mechanisms of esophagus (8). A conglomerated mass of heterogeneous with predominantly hypoechoic lymphnodes with intervening hyperechoic strands and foci on EUS appears to be characteristic of mediastinal tuberculosis (9). As the same in this study, multiple enlarged mediastinal lymph nodes fused with each other were seen in 66.7% patients. As we found in this study, dysphagia with or without retrosternal pain is the most common symptom and most lesions were located in the middle of esophagus, and these results are consistent with existing reports (9–14). Constitutional symptoms such as fever, weight loss and night sweats are nonspecific and difficult to distinguish. Under white light endoscopy, esophageal tuberculosis may present as an ulcerative, hyperplastic, granular lesion or extrinsic bulge (15). Most cases are misdiagnosed as cancer before surgical exploration (16, 17). As the same in our study, the presentations of symptoms, laboratory examinations, endoscopic findings combination with CT scan imaging of all cases are nonspecific. As a result, they do not provide a definitive diagnosis. Histocytopathological diagnosis is crucial before administration of appropriate therapy, and EUS-FNB has emerged as an excellent tool to diagnose esophageal tuberculosis (18).

The characteristic EUS morphology highly suggestive of esophageal tuberculosis, and EUS is a feasible diagnostic method. Firstly, we found that multiple enlarged mediastinal lymph nodes in close proximity to or fused with each other were presented as hypoechoic EUS areas which indicating lymphadenitis. Additionally, this study summarized the main features of esophageal tuberculosis, including heterogeneous hypoechoic masses in the thickened esophageal wall, interruption and breakage of the esophageal adventitia. Disappeared or ambiguous layers were observed in the esophageal wall as seen by other authors (19).

Esophageal tuberculosis should be distinguished from advanced esophageal carcinoma and leiomyoma. Thomas W. Rice et al. summaried the characteristics of esophageal cancer in 22,123 clinically staged patients, which showed patients were older (62 years) men (80%) (20). In our study, patients were all aged (range 59–79 years), in which condition malignant tumor may presented as a first priority.

Although esophageal tuberculosis is very similar to malignancy, a few details from the results of this study may indicate significant differences. First, disease duration (ranged 15 days to 4 months) of esophageal tuberculosis patients in this study is actually short. But in esophageal cancer patients, disease course is often much longer. Second, it was reported that esophageal cancer most involved the distal esophagus (73%), which counter to esophageal tuberculosis. As we found, the site of lesions were most located in the middle of esophagus. Besides, the laboratory examinations such as tuberculosis test and several tumor markers contribute to identifying tuberculosis and cancer. Most of all, histological diagnosis is crucial. In contrast to imaging alone, image guided biopsy is more reliable to establish a definite diagnosis. According to the results of Puri et al., 84.5% of the esophageal tuberculosis patients were diagnosed by pathology (21). In this study, EUS showed characteristic morphology of esophageal tuberculosis in all cases. EUS-FNB provided the pathological diagnosis of tuberculosis in four of six cases, increasing the possibility of a positive diagnosis of tuberculosis.

In conclusion, EUS has characteristic appearances depending upon esophageal wall and mediastinal lymph nodes. EUS-FNB is an excellent method to establish an accurate diagnosis of esophageal tuberculosis.

## Data Availability

The raw data supporting the conclusions of this article will be made available by the authors, without undue reservation.
